# Interprofessional simulation in a student community clinic: insights from an educational framework and contact theory

**DOI:** 10.1186/s41077-019-0106-9

**Published:** 2019-12-20

**Authors:** Susan Waller, Debra Nestel

**Affiliations:** 1 Monash Institute for Health and Clinical Education, Faculty of Medicine, Nursing and Health Sciences, Monash University, Building 13D, Room DG11, 35 Rainforest Walk, Clayton, VIC 3168 Australia; 2 Department of Rural & Indigenous Health, Monash University, Clayton, Australia

## Abstract

**Introduction:**

Simulation in community care is a relatively understudied area. In this paper, we report a qualitative evaluation of the Simulated Client Interprofessional Education (SCIPE) program in a community clinic for undergraduate health and social care students in a rural setting. We sought to explore the stakeholders’ perceptions and experiences of training for, and conduct of, a simulated client-based activity to support the development of collaborative practice of students. We used an educational framework (*presage, process, product–3P*) and *contact theory* to analyse the evaluation data and suggest improvement strategies.

**Methods:**

Data on *professional* characteristics was collected from facilitators, simulated client and students. Facilitators and simulated clients received training. Written evaluations were collected after training and after the simulated clinics. Purposively sampled facilitators, students and community partner agencies participated in individual semi-structured interviews to gain deeper insights into experiences.

**Results:**

Fourteen clinics involved 5 facilitators, 12 simulated clients and 40 students. Fifteen interviews were conducted. The SCIPE program led to perceived improvements in students’ communication and awareness of interprofessional collaboration. Participation in the program enabled students to experience a holistic approach to client interviewing and development of competency in collaborative goal setting. Further attention to *presage* and ability of facilitators to build positive *contact* conditions was identified. Coordination from a central site facilitated exchange and quality assurance for all elements of the program. Scoping of conditions of positive contact enabled a greater understanding of students’ and facilitators’ evaluation of the experience and constraints which would be modifiable for future improvement and sustainability.

**Discussion:**

Although the SCIPE program benefited students, the need for more explicit organisational engagement and support was revealed in interviews. The use of 3P and contact theory was helpful in identifying elements of the program for maintenance and development. Future research could follow students into practice to see if the behaviours are sustained and translated. Strengths included broad stakeholder involvement and immediate feedback. The key limitation was that the activity lacked explicit institutional support, facilitators required further training in briefing and the outcomes largely refer to participants’ perceptions and may not translate to practice.

## Introduction

Interprofessional collaborative practice and education are the cornerstones of safe patient care [1]. Immersive simulation scenarios promote collaboration between healthcare professionals and can lead to improved patient care [2–4]. IPE occurs when two or more professions (students) learn with, from and about each other to improve collaboration and the quality of care [5].

The value of interprofessional education (IPE) is that once healthcare students learn to work collaboratively, patient outcomes are likely to improve. Simulation is “a technique, not a technology, to replace or amplify real experiences with guided experiences, often immersive in nature, that evoke or replicate substantial aspects of the real world in a fully interactive fashion” [6]. Studies that have used simulation to promote interprofessional collaboration have reported short-term impact suggesting improved communication within and between healthcare professions [7–10]. Simulation provides participants with the freedom to make mistakes, learn from them and improve communication and processes of care.

### The SCIPE program

The Simulated Client Interprofessional Education (SCIPE) program was developed as part of a national funding program to deliver simulation-based education to rural and remote regions in Australia [11]. The primary objective of the SCIPE program was to offer interprofessional simulations that would support the work readiness of health professional students. Health professional students jointly interviewed simulated clients in consultation rooms equipped with audio-visual recording equipment (SimView). After the consultation, they participated in facilitator-led debriefing including the simulated client. The program was conducted under the auspices of a large independent community health service in regional Victoria, Latrobe Community Health Service (LCHS). The LCHS offers clinical placements to health professional students and through a Memorandum of Understanding with Monash University Department of Rural Health is afforded program organisational support. This paper builds on a preliminary study in which the program is fully described [12] and presents self-report data on the success of developing, delivering and evaluating simulated client-based activities for students from eleven health disciplines. Here, we used data from a subsequent implementation and apply an educational framework [13] and contact theory [14] to make meaning of the qualitative evaluation.

### Theoretical frameworks—3Ps and contact theory

First, in the educational framework, elements of learning are represented by *presage, process* and *product* (3Ps) [13]. The framework enables educators to consider all necessary elements of a learning activity and propose improvements where deficits are identified. See Table 1 for application to the SCIPE program. Liaw et al [15] used this framework to describe design and evaluation of interprofessional communication training using simulation to manage deteriorating patients. The 3P model enabled the identification of factors that enabled success in the initiative [15].

**Table 1:** Application of the SCIPE program to the presage, process and product educational framework

*Presage* is the relationship between the personal and situational elements of learning, the context. The context in the SCIPE program was that the simulation was conducted in a community health service with students from different disciplines. Facilitators and simulated clients were the ‘teachers’ in the activity so their personal characteristics and previous learning also influenced this dimension. *Process* includes approaches to learning. In the SCIPE program, facilitators and simulated clients trained in small groups and the activity was conducted in small groups where the training and learning with simulation were conducted with a shared structure. *Products* are the outcomes of learning. In the SCIPE program, these included trained simulated clients, clinic facilitators and health professional students who experienced interprofessional collaboration. This collaboration afforded students the opportunity to improve interviewing skills, learn more about each other’s roles, the programs of community health services and collaborative person-centred goal setting.	

Second, to understand the interpersonal interaction and learning that occurred in the SCIPE program, particularly in the simulated clinic, we posited the value of contact theory. Allport [14] formalized the theory, stating that intergroup contact would lead to reduced intergroup prejudice if the contact situation embodies the following four conditions:

1. Equal status between the groups in the situation;
2. Common goals;
3. No competition between the groups; and
4. Authority sanction for the contact.

Hewstone and Brown [16] identified further essential conditions for positive contact including participants:

1. Having positive expectations;
2. Joint work achieving success (cooperating rather than competing);
3. Having awareness of each other’s differences as well as similarities; and
4. Perceiving each other as typical members of the group.

In an IPE setting, this means that students are identified as *health professional* students rather than by their individual discipline [17], that they demonstrate interprofessional competencies described by O’Keefe et al. [18] for health professional students in Australia. This process of dual socialisation is supported by positive interprofessional interactions and may be carried over into the workplace if the culture supports that superordinate identity [19]. Positive contact conditions are needed such that both IPE experiences and practice settings are optimally interactive, egalitarian, cooperative and mutually supportive for all participants [16].

### Interprofessional simulation in community care

There is limited literature on interprofessional simulation in community or social care. The majority of the research on interprofessional simulation focuses on the clinical roles of healthcare professionals, and the physical health of the patient/client only [7, 8, 10, 20]. There are limited structured opportunities for students to interact with a variety of different professions to collaborate on care planning despite placement in multidisciplinary teams in community settings. Clinical placements afford such learning opportunities but simulation offers deliberate practice of collaborative competencies in a safer space. In the SCIPE program, students had the opportunity to learn about the community health setting and services offered to clients, as well as to learn *with*, *from* and *about* another profession.

## Methods

The initial SCIPE program was described in Taylor et al [12] and summarised here in Tables 1, 2 and 3. All participants completed evaluation forms in which they were invited to rate the degree to which learning objectives were met and the value of the educational methods. Numerical ratings and free-text data were collected. However, our interest in this paper was in seeking understanding of learning through analysis using 3P framework and contact theory of the free-text comments and the transcribed interviews.

**Table 2:**
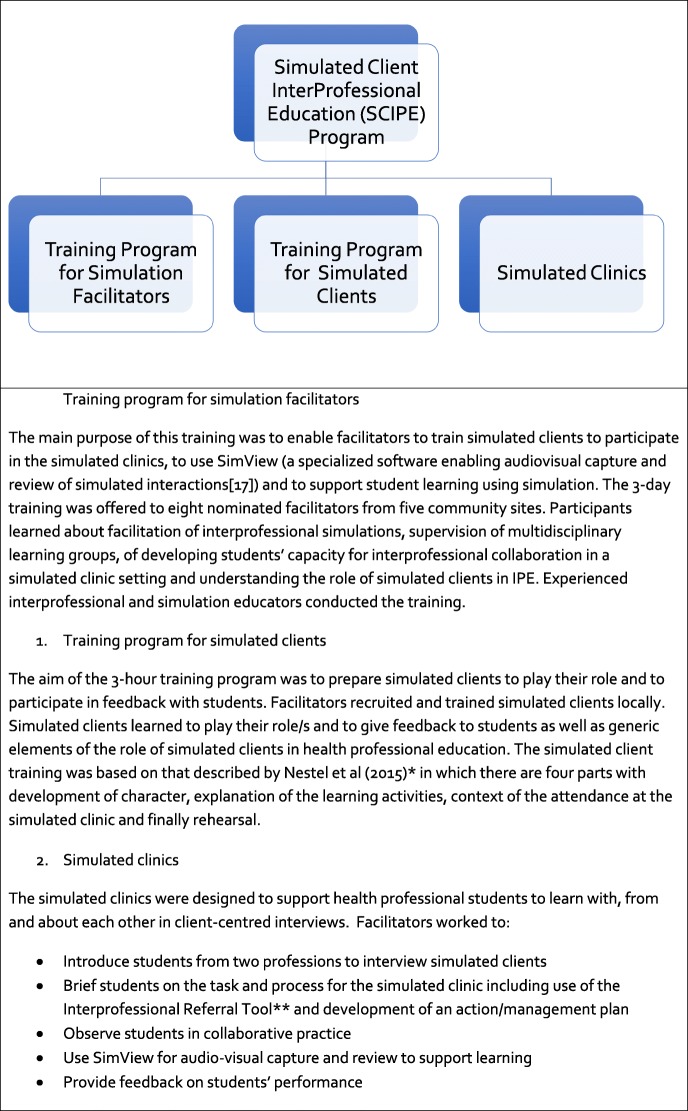
The SCIPE program components

*Nestel, D., Fleishman, C., and Bearman, M. (2015). Preparation: Developing scenarios and training for role portrayal. In D. Nestel and M. Bearman (Eds.), *Simulated Patient Methodology: Theory, Evidence and Practice* (pp. 63-70). West Sussex: John Wiley and Sons Ltd.

**The *Interprofessional Referral Tool* is a holistic intake tool, used in the community health service which enables a clinician to conduct a comprehensive initial assessment and develop a collaborative person-centred goal-directed management plan.

**Table 3:** Outline of the training program for facilitators

Program and time	Overview/activity	Learning outcomes
Interprofessional collaboration (IPC), 1 day (student-simulated clinic)	Basic introduction to interprofessional collaboration (IPC)	The facilitator will build their knowledge, skills and professional practice in IPC and apply it to project activities and to their everyday role
	IPC facilitation	
	IPC student workshop	
	IPC in the workplace—includes giving constructive feedback	
Implementing a simulated clinic (training for facilitators), 1 day	Principles of simulation drawing on the NHET-Sim program	Following this training, the facilitator will develop local simulated client scenarios; assist in the training of local simulated clients; plan, organise and conduct simulated clinics; prepare students from two different disciplines to undertake the simulated clinic experience and provide support and constructive criticism to all participants
	Case scenario introduction	
	Case scenario development	
	Conducting a simulated clinic	
	Training simulated clients	
	Supporting simulated clients	
	Giving constructive feedback	
	Briefing and debriefing	
Training program for volunteer ‘simulated clients’, 3 h	Becoming a simulated client	Following training the volunteer clients will learn specific simulated client scenarios; Undertake the role of the simulated client and provide constructive feedback to students
	Learning your role	
	Adapting and responding to students’ questions	
	Giving constructive feedback	
SimView Information technology, 3 h	Operating the SimView system	The facilitator will be proficient in using SimView to capture and edit the simulated clinic audio-visual data; facilitator will access the MUDRH website to upload the simulated client scenarios to share with other partners; to seek and answer questions and to keep up to date with project developments
	Editing, saving, storing and sharing video files	
	Accessing the MUDRH website and interprofessional resources	
	Downloading and accessing scenarios for simulated clients	
	Facilitators—chat room	
	Simulated client program—frequently asked questions	

Purposively sampled stakeholders (facilitators, students and community health agency partners) were interviewed after the SCIPE program. Although we had intended to interview simulated clients, the logistics of the project and funding constraints prevented their involvement. Interviews were conducted by a research assistant with experience of qualitative interviewing. The topic guides were developed with the project goals in mind (Appendices 1 and 2). Interviews were conducted face to face or by phone at a time convenient to the interviewee. Recordings were made and transcribed. Interviewees were offered but did not take up the opportunities for transcription validation.

Researchers read and reread transcripts, manually coded individually and then together discussed with each other to develop a final representation of themes emergent from the data. Themes were then aligned with the 3P framework and contact theory tenets.

An ethics application was submitted to the Monash University Human Research Ethics Committee and was given approval.

## Results

### Facilitators

Five facilitators participated in the SCIPE program of whom four were females and one male, three were nurses, one was a podiatrist and one a health promotion officer. They all worked for a public healthcare service, were all born in Australia and had no prior training in interprofessional or simulation-based education.

### Simulated clients

Twelve simulated clients participated in the SCIPE program. Eleven were female and one male, aged from 55 years to 83 years old, with a mean age of 66 years. Seven were born in Australia. One simulated client had prior experience as a simulated patient.

### Students

Forty students participated in the community clinics. There were 22 females and 18 males, aged from 20 to 52 years, with a mean age of 27 years. The majority of students were born in Australia (*n* = 32; 80%), students were studying medicine (*n* = 18; 45%), nursing, (*n* = 13; 32%), with the balance of students coming from aboriginal health liaison, physiotherapy, exercise physiology, allied health assistance, occupational therapy and podiatry. Twenty-seven students were from Monash University while the remaining 13 students came from 6 other universities, a technical college and a training provider.

### Thematic analysis aligned with the 3P framework and contact theory elements

Participants were asked to reflect on the training and simulation clinic experience and to respond to questions about what worked well and what they found challenging**.**

Themes in the analysis were aligned to elements of the 3P learning framework:

- Providing opportunities for IPE (presage)
- Training for simulation facilitators (presage/process)
- Developing reflective practice and an interprofessional perspective (process)
- Implementing simulations (process)
  - Working with simulated clients
  - Briefing
  - Providing feedback/debriefing
  - Using audio-visual capture and review (SimView)
  - Authentic practice
  - Accessing and arranging resources
  - Integrating the interprofessional referral tool (IRT)
- Building collaborative competencies (product)
- Embedding the SCIPE program (product)
- Shifting culture and supporting innovation (product)

Themes are demonstrated as follows with illustrative quotes from participants and analysis of their significance for the evaluation, improvement and sustainability.

### Providing opportunities for IPE

Providing opportunities for IPE in extended settings was recognised as valuable for student learning in these placements.

It’s a real plus for a small hospital like us to have something like this. Lots of the time it’s the bigger places who get asked, not small hospitals like us. (Partner agency)

Such sentiments expressed by partner agencies are important with respect to sustainability since all programs require local champions to keep them running when project funding is expended.

### Training for simulation facilitators

The training was good but what we really needed to do was see a real simulation with students in practice, because it all made sense when we saw that. (Facilitator)

In training programs, it is helpful to have examples of what the expected practice is to be as it enables the participants to use less imagination and focus on acquiring the new skills they will require to implement a successful simulation.

### Developing reflective practice and an interprofessional perspective

Seeing the students change in that short period of time, so they can reflect on what has happened and also learn from that. (Facilitator)

Where the simulation fits on placement is worthwhile considering as for some students it allows a safe space to enter placement. It may also be used to confirm that increased competency at the end of a placement.

We did have one student who performed quite poorly in his first interview and then he has had six weeks of placement in between his next interview and that second interview was amazingly better. (Facilitator)

### Implementing simulations

#### Working with simulated clients

The volunteers are amazing and you absolutely come out of there feeling like you have spoken to a real patient with ongoing issues. (Student)

Authenticity was experienced in the activity by students, with simulated clients representing real scenarios from the local community health context.

The program seemed to have prepared these simulated clients well for the setting in which they were working. However, facilitators expressed a challenge with the simulated clients continued commitment and recognised that recruitment would have to continue for the activity to be sustainable.

Keeping your simulated patients [clients] engaged and having enough that you are flexible - at the end we had limited sim patients and probably if we were ongoing we would be recruiting. (Facilitator)

The program was innovative in its application of simulated clients in a community health setting. There are few documented accounts of simulated clients in this context.

### Briefing

The quality of the simulation educational experience usually resides with the briefing. That is, setting the tone, clear objectives and structures are critical for success. It appears that this is an area for development in training facilitators.

We were not given any actual data about what should be included in the briefing. We had to come up with our own which probably was not in keeping with what was wanted in the briefing. (Facilitator)

Although part of the process of learning, the briefing is also an element of presage as it *sets the stage* of the learning activity and explores the understanding of the students and facilitators in the simulation that follows. Lack of confidence at this stage by the facilitator may have impacted on the learning experience of the students but also does identify an area to strengthen in the training and support for facilitators.

### Providing feedback/debriefing

Considering process, feedback and debriefing are an essential component of a simulation [21–23]

The discussion afterwards, after the interview, was helpful. And the discussion with the medical student afterwards was good to see what he thought. (Student)

Getting feedback off the person you are interviewing, as well as facilitators and students worked well. (Student)

These comments are illustrative of the importance of feedback and debriefing after a simulation [21–23]. Skilled facilitators can manage this process effectively and it is often considered when the “real” learning occurs. However, skilled facilitation takes time to develop. The students’ responses suggest the facilitation was effective. Facilitating feedback from others involved in the scenario is also critical and this seemed to take place with students learning from feedback from the simulated client but also from each other. Including the “unique” perspective of the simulated client was valued and the program applauded for this feature.

### Using audiovisual capture and review (use of SimView)

There definitely exists unease with some participants around the use of video capture and replay.

The video feedback thing at the end was just a bit awkward. (Student)

Watching themselves talking on video was just weird. It is not something you do often, so it is weird to see yourself in third person. (Student)

Facilitators could see the value of the real-time viewing afforded by SimView.

Provides a safe environment, able to step in if needed. (Facilitator)

Facilitators able to be out of the room and not hovering over the top of the role-play – greater learning opportunity, more realistic. (Facilitator)

The use of audiovisual equipment to augment the feedback process requires deliberate practice. Specific technology skills have been identified and it was not apparent that facilitators were supported in this way in the use of SimView. Skills for effective use include recording the whole scenario but selecting specific segments for discussion (15–45 s), that annotation should be used, facilitators should preview the clips if time permits, discussion usually precedes the showing of the clip such that participants are orientated to what they will be viewing.

The equipment - very very frustrating- had to cancel 2 or 3 different clinics. (Facilitator)

Ensuring equipment is working in advance is essential, and it was apparent that SimView was not always working. This can negatively impact all participants’ attitudes towards the program. Using technology to add value to a learning experience relies on a functioning system and operators have adequate knowledge and confidence to troubleshoot. Facilitators require further development in this area if they are to continue to use this technology.

### Authentic practice

Taking histories with another person- it is really unrealistic because it never happens- especially for an hour. (Student)

It is important that the scenarios are appropriately framed; that the briefing explicitly identifies the reasoning for any departure from standard practice. The value of interviewing with a student from another discipline was in augmenting the opportunity for students to learn further about another health professional role and perspective. There was also the deliberate purpose of performing a task together that was not discipline-specific but emphasised common collaborative competencies such as goal setting.

### Accessing and arranging resources

The simulated clinics required physical resources which seemed to function effectively in this program. It is important in any simulation activity that attention is paid to the layout of the room, privacy, seating arrangements, location of the camera and many simulation educators insist on a separate space for briefing and debriefing to create clear separation in from the simulation where emotions can be very high. The investment in audio-visual resources for facilities was appreciated by partner agencies for its flexibility and multi-purpose potential. The rooms used are regular consultation rooms and this added authenticity to the activity.

Physical environment is really important. Location and size of the rooms. (Facilitator)

We are going to be left with equipment we can use for lots of things. (Partner agency)

It took over a room also which is another resource. (Partner agency)

### Integrating the Interprofessional Referral Tool

The following student found that when completing the tool, there was too many indications for referral that may have not have covered all areas in the simulated client presentation.

The checklist of what you had to do. It was really poorly written and it oversupplied services to the patient [client] and then missed certain things. Clinicians need the opportunity to ask more open-ended questions and re-assess the patients rather than go through a checklist. (Student)

This perspective was also demonstrated by this facilitator.

A lot of students were baffled with the actual interprofessional referral tool. It is a bit restrictive. (Facilitator)

In contrast, this student found the format useful and relevant.

We had some guidelines to follow which helped so we weren’t thrown in there thinking, what am I doing? (Student)

The positive evaluation of the activity by students was reflected in the insight expressed below. This quote demonstrates the value recognised in interprofessional learning for better practice.

Should be addressed earlier in the course, as soon as we start clinical practice. There are a lot of doctors who don't know the role of physio, OT, speech pathologists, so would be beneficial for learning. (Student)

The workload was significant for the participants, facilitators and students. Unless educational activities form part of hurdle assessments/curriculum then students may be unwilling to engage. Further, workplaces need to support facilitators in undertaking such work. It is apparent that there was at least the perception that this was being squeezed into an already busy schedule for community health service and education staff.

Set up time was burdensome, we had no administrative support. (Facilitator)

Further, part-time funded project support positions meant that help was not always available when needed. Therefore, the deficit of the positive contact condition, institutional support, was also perceived by facilitators.

### Building collaborative competencies

Considering conditions for positive contact, facilitators and students identified that learning occurred related to acknowledgement of varying roles and benefits of working together on a common goal. Another element of positive contact was also cited in the quotes below with students recognising a fellow student as an authentic member of that discipline group. They developed an increased understanding of discipline differences and how to work together for better patient care.

The main thing was just the awareness of nursing thought process and concepts. It now makes more sense when you discuss patients with them. (Student)

I was there with a physio student. I actually learned a lot about what his capabilities would be as a professional. I was not too aware of it as I have not had much exposure to the actual ins and outs of what physios do. (Student)

Students really get the gist of how collaboration can benefit and how it can streamline their time. (Facilitator)

I’ve never worked with a different professional, it made me feel more confident to talk to them and apply more appropriate treatment to the patient. (Student)

### Embedding the SCIPE program

The challenge to sustainability for some students and also facilitators was directly attributable to an important deficit in positive contact conditions. If students perceive that the activity is not supported by the higher education provider (not assessable) then they are unlikely to engage in the interaction with the same focus.

I think some of the students might be hesitant to do it because it is not part of their core curriculum. It really comes back to the time thing. (Facilitator)

It is much more practical than a lot of the other things we do on the course but it is not viewed as important by the university – it is not a compulsory learning session. I would be more than happy to have large fractions of my current course replaced by interprofessional learning. (Student)

This deficit also impacted the workload experienced by facilitators as recruitment was time-consuming and sometimes difficult with students not being directed to participate by course requirements but invited by facilitators.

The recruiting of the students was the most time consuming. (Facilitator)

For some facilitators, there was some dissonance between their traditional role as nurse educators in the health service and as facilitators in the project.

It has meant that staff development nurses have been doing this instead of other educational things. (Partner agency)

### Shifting culture and supporting innovation

Contributing to the building of a collaborative culture is perhaps the most exciting element of the evaluation data because it hints at embracing innovation and thinking about new ways of doing things. Although there were many challenges with the SCIPE program, what is important is that a sound product has emerged that has scope for application more broadly and that the settings in which it is valuable are more open to new methods.

I think it’s very progressive and [an] exciting thing to be on and I can see great benefits in the process. It is becoming part of our overall vision around learning as an organization. (Partner agency)

It has given us support in being more innovative and progressive with the education of students – it gives you the backing and the push to achieve something that you would otherwise not normally go there. (Partner agency)

## Discussion

Overall, students and facilitators responded positively to participation in the program. Interprofessional simulation of an intake interview to devise a collaborative client-centred management plan can strengthen the clinical placement learning and support the work readiness of student participants. Several aspects of the evaluation results offer improvement indications and requirements for sustainability. These may constructively be discussed using the 3P framework to identify components of the program for strengthening.

The evaluation indicated that there is a need to strengthen the development of facilitation of students’ learning about interprofessional collaborative practice. Understanding the educational framework that underpins interprofessional activities also required further development. The *presage* to learning in this activity related to previous experience and skills of students and facilitators. Feedback and debriefing require continual development and reinforcement to promote educator confidence. Training for simulated clients would include developing an understanding of simulation as an educational method and simulated client roles for demonstrating interprofessional practice.

Some facilitators reported feeling stressed at the beginning. In terms of workload, the beginning was difficult due to the setting up procedures and recruiting of students was sometimes difficult.

In order to maintain their strengths, facilitators must be able to supervise the learning of a small interprofessional group of students/faculty, develop their capacities for interprofessional collaboration in a simulated clinic setting and understand the role of simulated clients in health professional education. Such facilitator competencies require continuous training and ought not to be expected to be developed in discipline-specific clinical educators. Support by health services for specific training in interprofessional facilitation will enable sustainability and attributed to the quality of the program.

The facilitators were impressed with their simulated clients. However, reported difficulties in keeping them engaged and having enough clients so that they could be flexible.

Volunteer-simulated clients functioned effectively in supporting students and were able to create an authentic interaction of a complex scenario. Relying on volunteers is satisfactory for a pilot study but may need reviewing for sustainability. There is also a different relationship with volunteers than with contracted simulated clients. However, potentially in a rural/regional setting, connection with volunteers may be stronger and continuing engagement will require dedicated ongoing development and explicit valuing.

Partner agencies reported that time and money to run the program and the fact that the project staff were part-time were key challenges. There was a perception that the program was very progressive and an exciting program to join. They felt that the workload was considerable from an organizational perspective, and they had to pull staff from other areas to run the program. Although the program took up resources, such as rooms, the partner agencies appreciated being able to keep the equipment which they will use for a number of different programs.

Considering the process of supporting IPE, facilitators require signposting of key elements of written scenarios and how to make these explicit in training for simulated clients.

Training for simulated clients would include developing an understanding of simulation as an educational method and simulated client roles for demonstrating interprofessional practice. This training would include a review of basic educational principles and simulated client training using the read, review and rehearse process. Evaluation of the training of simulated clients revealed that there is a need to refresh regularly an understanding of the role and to review and practice basic principles of feedback.

Compared with other types of simulations, the scenarios were quite long; an average of 38 min. Although this is valuable, for students new to interprofessional collaborative practice, it may be too long to be in a continuous activity without feedback. It was not clear the extent to which the simulation could or was “interrupted”, and there was no evidence of techniques such as “pause and discuss” being used. Scaffolding learners across the length of the student-client interaction may result in more valuable outcomes. The variability in briefing and debriefing also suggests that tighter guidelines around program intent.

When SimView works, it is useful for several reasons but particularly because it enables remote and synchronous observation leaving participants in the simulation room alone and also enables the feedback to be augmented by audio-visual illustration. Ensuring that students and facilitators understand the purpose of the recording and playback with an emphasis on supporting constructive feedback in a safe environment may mitigate feelings of discomfort experienced by some students.

The IRT had a mixed response and appears to require further work in content and orientation to use. It is possible that the use of the IRT was too advanced for the level of students’ capability. Flexible use and adaptability of the tool may require greater clinical experience. However, use of a holistic tool which is not discipline-specific but affords a platform on which to develop a client-centred plan was recognised by students and facilitators to support the development of collaborative competencies.

Improvement would require reviewing the educational methods and resources used across the training program for facilitators and simulated clients. In particular, reviewing the training resources and experiential activities in the facilitator program were found as essential to the development of facilitation competencies. The process could be enhanced by including video clips of student-client interactions to help facilitators reflect on their capacity to align learning objectives with the activity. The development of a targeted simulated clinic session guide that specifies the content of briefings and offers more structure for feedback and debriefing would support ongoing facilitator competence.

Facilitators thought that it would have been beneficial to see a simulated clinic first before receiving the training so they could understand it better. They also reported that there was too long a gap between training and when the clinics started, so they forgot some of the information. Facilitators needed more information on what to include in the briefing with the students. They also thought that the physical environment was very important and particularly the location and size of the rooms.

Positive contact conditions supported the process. Students understood the activity as having a common positive goal of devising a collaborative care plan. Students in interprofessional pairs and in debriefing afterwards recognised each other as authentic members of their respective disciplines. They were able to recognise shared tasks and discipline-specific tasks during the activity.

Some students found being filmed quite “daunting” and watching the recordings of their interviews “awkward”. They appreciated the use of simulation and found the context and conduct of the presentation and interview with the simulated client authentic and instructive. However, doing the interview in pairs was criticised by some students as unrealistic and facilitators required well-developed briefing skills to explain the approach as integral to the interprofessional experience.

The IRT was not generally well received by the more experienced students, who found it ‘restrictive’; however, some students found it helpful to guide them through the interview process. Students found the feedback from simulated clients, facilitators and peers to be a positive learning tool.

The product of the training and activity was that the simulated clinics were a beneficial experience for students, and they would recommend their peers also participate. Students learned about the roles of other professions and the program enhanced their ability to cooperate, manage and plan together; building their interprofessional collaborative competence. Some students reporting that the program made them feel more confident in talking to other professionals, able to assist in applying more appropriate treatment for the patient and raised awareness of the roles of other professionals. Conducting a holistic assessment with a simulated client enabled a greater understanding of the breadth of services offered in a community health centre and improved collaborative person-centred goal setting.

The simulated clients provided a positive experience. It was felt that this program was more practical than many other tasks students do at university. Students would appreciate these tasks to occur earlier in their courses and to have more tasks with a focus on interprofessional collaboration.

Deficits in positive contact conditions were also reported. It was suggested that students may not be willing to participate because the program is not part of their core curriculum and that because they have so many other requirements to meet this is not a priority. It was also felt by some students that the interview is unrealistic because it is not likely that they would be interviewing a patient for an hour with another health professional. These deficits in the perception of authority support and an expectation of positive outcomes are essential to address to improve the program.

### Limitations of the evaluation

There were several limitations to the evaluation including the phrasing of some learning objectives in terms that were not measurable (e.g. replace “understand” with “discuss” or “describe”). The evaluation relies on self-report of practice and so does not address the objective transfer of practice. Finally, changes were not actioned during the life of the program so that changes could not be made in action.

### Strengths of evaluation

Multiple stakeholders and data types enabled triangulation. Evaluation by facilitators, simulated clients, students and partner agencies afforded a comprehensive evaluation but would have been strengthened by interview data from the simulated clients. Evaluation of transition of competencies into practice would be recommended in future evaluations.

## Conclusion

Using the *3P framework* and *positive contact* elements to retrospectively analyse evaluation from a simulation training and clinic activity has enabled a fuller understanding of strengths and challenges experienced by participants. Findings will afford indicators for improvement and sustainability of the activity. The SCIPE program was successfully implemented across a wide geographical area with coordination from one site despite significant challenges.

Students reported increased knowledge, attitudes and skills in interprofessional collaborative practice. Further evaluation needs to be undertaken to see if the actual practice is changed.

The continued investment by health services and higher education providers in training programs for facilitators and simulated clients is essential for the success of the program. Although a pool of facilitators and simulated clients has been developed, they will need ongoing support to maintain their skills. It is likely that additional facilitators and simulated clients will need to be trained. The value of local champions as evidenced in this program is important for sustainability and explicit value attributed by the health service management for participation by clinician facilitators and student recruitment. Administrative support is necessary to coordinate the program and resources. There is also a need for recognition by the university program and value given to participation in the students’ courses. Ongoing evaluation of the sustained activity will be important for future development and it is recommended that elements of the 3P learning framework inform this, underpinned by attention to positive contact conditions with the focus to shift from simply quality assurance to transfer of learning to the practice settings.

## Data Availability

N/A

## Appendix 1

### Individual interviews with facilitators

This is an example of the topic guide for facilitators. The guide for students complemented this one.

1. Having completed the SCIPE program, what are your thoughts and feelings about it?
2. Was there any standout experience? If so, what was it? Why?
3. Was there any negative experience? If so, what was it? Why?
4. How did SCIPE help your simulation and interprofessional education practice?
  - Can you give a specific example of something that you will change/have changed within your practice?
5. Were there any ways in which you felt SCIPE was unhelpful or deficient with respect to your simulation education practice?
6. Was there content that you think we should have included?
7. What are your reactions to the workload of the SCIPE program?
8. How successful was the train-the-trainer program in preparing you to teach students in the SCC sessions?
9. What do you think are the facilitating factors for the SCIPE program?
10. What do you think are the barriers to the SCIPE program?
11. To what extent did Sim View support student learning?
12. What was it like to use?
13. One of the goals of the SCIPE program was to build a community of practice of teachers/facilitators in simulation-based interprofessional educators. To what extent do you feel part of a community? Did the SCIPE program develop your local community of practice? In what ways?
14. What are your views on the role of interprofessional simulation-based education? Has the SCIPE Program influenced your thinking? If so, in what ways?
15. What do you believe are the most important elements of strategic planning in simulation-based education for rural locations?
16. Is there anything else you would like to share with us about the SCIPE program?

## Appendix 2

### Individual interviews with partner agencies

1. Having participated in the SCIPE program, what are your thoughts and feelings about it?
2. Was there any standout experience? If so, what was it? Why?
3. Was there any negative experience? If so, what was it? Why?
4. How has the SCIPE program helped your practice?
  - Can you give a specific example of something that you will change/have changed within your practice?
5. Were there any ways in which you thought the SCIPE program was unhelpful or deficient with respect to your practice?
6. Was there content that you think we should have included?
7. What are your reactions to the workload of the SCIPE program?
8. What do you think are the facilitating factors for the SCIPE program?
9. What do you think are the barriers for the SCIPE program?
10. In what ways has SimView been useful to your practice?
11. Has it been unhelpful?
12. What was it like to use?
13. One of the goals of the SCIPE program was to build a community of practice of teachers/facilitators in simulation-based interprofessional educators. To what extent do you feel part of a community?
14. What are your views on the role of interprofessional simulation-based education? Has the SCIPE program influenced your thinking? If so, in what ways?
15. What do you believe are the most important elements of strategic planning in simulation-based education for rural locations?
16. Is there anything else you would like to share with us about the SCIPE program?
